# Exploratory Psychometric Assessment of the Endler Multidimensional Anxiety Scales in Romanian Hemodialysis Patients: Reliability, Convergent Validity, and Domain-Level Structure

**DOI:** 10.3390/medicina62040694

**Published:** 2026-04-04

**Authors:** Adriana-Luciana Luca, Felicia Militaru, Virginia Maria Rădulescu, Cristina Mariana Văduva, Daniela Teodora Maria, Mădălina Iuliana Mușat, Ion Udriștoiu, Eugen Moța

**Affiliations:** 1 U.M.F. Doctoral School Craiova, University of Medicine and Pharmacy of Craiova, 200349 Craiova, Romania; eugenmota@yahoo.com; 2 Department of Psychiatry, University of Medicine and Pharmacy of Craiova, 200349 Craiova, Romania; felicia.militaru@umfcv.ro (F.M.); ion.udristoiu@umfcv.ro (I.U.); 3 Department of Medical Informatics and Biostatistics, University of Medicine and Pharmacy of Craiova, 200349 Craiova, Romania; 4 Hemodialysis Center, Emergency County Hospital of Craiova, 200642 Craiova, Romania; cristina_vaduva2002@yahoo.com; 5 Department of Nephrology, University of Medicine and Pharmacy of Craiova, 200349 Craiova, Romania; daniela.maria@umfcv.ro; 6 Experimental Research Centre for Normal and Pathological Aging, University of Medicine and Pharmacy of Craiova, 200349 Craiova, Romania; madalina.musat@umfcv.ro; 7 Department of Scientific Research Methodology, University of Medicine and Pharmacy of Craiova, 200349 Craiova, Romania

**Keywords:** chronic kidney disease, hemodialysis, multidimensional anxiety, state–trait anxiety, perceived anxiety, EMAS, psychometric assessment

## Abstract

*Background and Objectives*: Chronic kidney disease (CKD) is an increasingly important global health challenge and is frequently accompanied by psychiatric symptoms, including anxiety. A multidimensional assessment of anxiety in hemodialysis (HD) using the Endler Multidimensional Anxiety Scales (EMAS) has not, to our knowledge, been previously reported. We aim to evaluate the reliability, convergent validity, and exploratory domain-level structure of EMAS in HD patients treated at a dialysis center in Craiova, Romania. *Materials and Methods*: A total of 103 HD patients underwent clinical and sociodemographic/socioeconomic profiling, cognitive screening using the Mini-Mental State Examination (MMSE), and EMAS administration at two time points (4-week interval) for test–retest evaluation. The anxiety subscale of the Depression, Anxiety, and Stress Scale-21R (DASS-21R) was administered to assess convergent validity. Internal consistency (Cronbach’s α), temporal stability (test–retest correlations and intraclass correlation coefficients), and convergent validity (Pearson correlations) were computed. Exploratory factor analyses were conducted on EMAS domain scores (state, trait, and perceived anxiety domains) as an exploratory structural check. *Results*: EMAS state and trait anxiety scores were higher in women than in men, while perceived anxiety showed a more heterogeneous pattern across dimensions. Total state anxiety increased with age, particularly after 50 years. Domain-level internal consistency was good for state and acceptable for trait components (standardized α ≈ 0.84 and 0.78 across administrations), whereas perceived anxiety domains showed low cross-domain coherence, consistent with context-specific appraisal. The DASS-21R anxiety subscale showed good internal consistency (α = 0.863). Convergent validity analyses indicated small, domain-specific associations between EMAS scores and DASS-21R anxiety. Domain-level EFA supported a theoretically coherent pattern in which state and trait domains clustered distinctly, while perceived anxiety domains formed a partially separable factor; this pattern was broadly consistent across both administrations. *Conclusions*: In this HD cohort, EMAS demonstrated good reliability and limited but domain-specific evidence of convergent validity, and exploratory domain-level analyses supported its multidimensional organization. Further studies with larger samples are warranted for item-level structural testing and to inform feasibility-oriented shortening for potential clinical use.

## 1. Introduction

Hemodialysis (HD), the standard treatment for end-stage chronic kidney disease (CKD), imposes a substantial physical and psychosocial burden, requiring strict adherence to treatment schedules, dietary and fluid restrictions, and repeated exposure to invasive procedures. These constraints significantly affect patients’ quality of life and contribute to the development of psychological distress.

Anxiety is among the most prevalent and clinically relevant psychiatric symptoms in HD patients, with a well-documented negative impact on quality of life, treatment adherence, and clinical outcomes [1,2,3,4]. The Kidney Disease: Improving Global Outcomes (KDIGO) Controversies Conference in Supportive Care has identified anxiety symptoms and disorders as a key research priority in this population [5].

The HD setting exposes patients to multiple and recurrent stressors, including uncertainty regarding disease progression, frequent medical interventions, perceived loss of autonomy, and reduced social and occupational participation. In parallel, symptom burden—such as fatigue, sleep disturbances, pruritus, and pain—may amplify emotional distress and sustain anticipatory anxiety. These interacting factors support the need for a multidimensional conceptualization of anxiety that distinguishes between transient emotional states, stable vulnerability traits, and perceived anxiety in context [6,7,8,9].

Anxiety in HD patients has been associated with reduced quality of life, poor treatment adherence, increased hospitalization, and higher mortality risk, independently of other clinical factors [2,10,11]. Despite its clinical relevance, the assessment of anxiety in this population remains methodologically limited.

Commonly used instruments, such as the State–Trait Anxiety Inventory (STAI), the Hospital Anxiety and Depression Scale (HADS), and the Beck Anxiety Inventory (BAI), primarily provide global or partially differentiated measures of anxiety [5,12,13]. While useful for screening purposes, these tools offer limited insight into the interaction between situational, dispositional, and cognitive components of anxiety.

The Endler Multidimensional Anxiety Scales (EMAS) are based on an interactional model of anxiety, distinguishing between state anxiety, trait anxiety across situational domains, and perceived anxiety. This framework allows a more detailed evaluation of anxiety responses, which may be particularly relevant in HD patients, where psychological responses are shaped by both individual predispositions and treatment-related stressors.

In Romania, data on anxiety in HD patients remain limited, highlighting the need for context-specific research [4,14,15]. Furthermore, the psychometric performance of EMAS has not been previously evaluated in this clinical population.

Therefore, the aim of the present study was to assess the reliability, convergent validity, and exploratory domain-level structure of EMAS in Romanian HD patients, providing preliminary evidence for its applicability as a multidimensional research instrument in this setting.

Although brief instruments are often preferred for routine screening due to lower respondent burden, they typically yield global symptom scores and do not disentangle state-, trait-, and context-specific components of anxiety. Given our objective to evaluate the multidimensional anxiety model, EMAS was selected as a research instrument. We acknowledge the increased respondent burden and consider this aspect a limitation; however, the comprehensive administration of EMAS was necessary to preserve the full theoretical structure of the model and to support future development of shorter, clinically feasible versions. The aim of this study is to evaluate the psychometric performance and structural characteristics of the EMAS in a cohort of HD patients and to assess its convergent validity using an established anxiety measure.

## 2. Materials and Methods

### 2.1. Study Design and Participants

This observational, single-center, two-stage psychometric study was conducted at the DIAVERUM Nephrology and Dialysis Center in Craiova, Romania, over a two-year data-collection window (December 2021–November 2023) and included a test–retest component to estimate temporal reliability.

**Figure 1:**
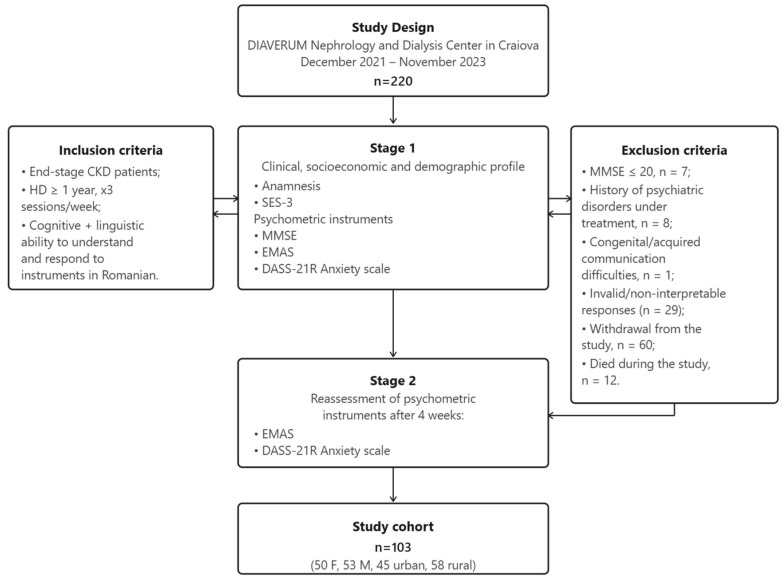
Study design and data collection process. Flowchart illustrating study period, inclusion and exclusion criteria, sample characteristics, and data collection methods applied to 103 HD patients (50 females, 53 males; age range: 33–84 years) at the DIAVERUM Nephrology and Dialysis Center in Craiova (December 2021–November 2023) (Adobe InDesign v20.5 64-bit).

The study cohort included 103 participants, 50 women (48.5%) and 53 men (51.5%); 45 (43.7%) were from urban areas and 58 (56.3%) from rural areas; participants were aged between 33 and 84 years (Figure 1).

### 2.2. Inclusion and Exclusion Criteria

Inclusion criteria: Patients with end-stage CKD undergoing HD for at least one year, attending three sessions per week, and possessing the cognitive and linguistic ability to understand and respond to the instruments in Romanian.

Exclusion criteria: Patients scoring ≤ 20 on the MMSE, those with psychiatric diagnoses under pharmacological treatment, individuals with communication or comprehension impairments, those exhibiting clearly implausible and non-directional response patterns across the majority of EMAS domains at both administrations, identified by the administering clinician upon physical receipt of the questionnaires prior to data entry, and deemed inconsistent with any plausible psychological trajectory over the 4-week interval (*n* = 29), or those who voluntarily withdrew from participation.

### 2.3. Data Collection Process

After obtaining written informed consent from each participant, data collection proceeded in two primary stages:

(a) Clinical, socioeconomic, and demographic profiling.
  Customized anamnesis was conducted to collect data on sex, age, residential environment, medical history, duration and frequency of HD treatment, and social/family support. Socioeconomic status (SES) was assessed using the SES-3 questionnaire and reported descriptively; it was not incorporated into inferential analyses.
(b) Administration of standardized psychometric instruments.

All participants completed the following:

- MMSE: used at baseline to identify potential neurocognitive impairments that could compromise the validity of psychometric assessments and was not included in the test–retest procedure.
- EMAS: administered twice, four weeks apart, following the standardized manual. To ensure data quality, response consistency between the two EMAS administrations was evaluated at the domain level by the administering clinician at the time of questionnaire collection, prior to data entry. Cases displaying clearly implausible and non-directional response patterns—characterized by large, incoherent variations across the majority of EMAS domains that could not be attributed to any plausible clinical or psychological change over the 4-week interval—were excluded from the analytical sample (*n* = 29). These cases were identified at the point of physical receipt of the completed questionnaires; the original forms were not retained for further analysis, and retrospective quantification of their T1–T2 discrepancies was therefore not feasible. Although a formal quantitative threshold was not pre-specified at the time of data collection, future studies should define response inconsistency using reproducible criteria, such as T-score differences exceeding ±2 standard deviations across multiple domains or non-directional variability patterns exceeding predefined percentile thresholds. The second administration was planned specifically to estimate test–retest reliability, which assumes temporal stability of the measured construct, rather than to model short-term symptom dynamics, which would require more frequent repeated measurements.
- Depression, Anxiety and Stress Scale-21R (DASS-21R), specifically the anxiety subscale, was included to support the results obtained with the EMAS, given the absence of previous EMAS-based studies in CKD or HD populations.

Because the assessment battery was time-demanding, respondent burden was anticipated, and participants were free to discontinue participation at any time; in practice, voluntary withdrawal due to fatigue or lengthy testing time represented the most frequent non-eligibility reason (*n* = 60).

### 2.4. Variables and Measurements

#### 2.4.1. Cognitive Function Assessment

Cognitive deficits were assessed using the MMSE, a widely standardized tool developed by Marshall Folstein in 1975 [16]. The MMSE evaluates global cognitive function across domains such as orientation, memory, attention, calculation, language, and abstract reasoning. Scores range from 0 to 30. A cut-off of ≤20 was applied as an exclusion criterion in order to exclude patients with moderate-to-severe cognitive impairment while retaining those with mild impairment who retained the ability to reliably complete psychometric assessments.

#### 2.4.2. Multidimensional Anxiety Assessment

Anxiety was assessed using the EMAS—a complex psychometric tool comprising 85 items—which was adapted and standardized for the Romanian population under license. The EMAS distinguishes between state anxiety (EMAS-S), trait anxiety (EMAS-T), and perceived anxiety (EMAS-P).

EMAS-S: Contains 20 items evaluating temporary, modifiable anxiety at the time of testing, divided into (1) cognitive anxiety (EMAS-S-C)—feelings of insecurity, helplessness, concentration difficulties, inadequacy, and (2) emotional–physiological anxiety (EMAS-S-EF)—intrapsychic tension and somatic symptoms such as trembling, sweating, irregular heartbeat, throat tightness, or dry mouth. Items are rated on a 5-point Likert scale from 1 (“not at all”) to 5 (“very much”), generating three scores: EMAS-S-C, EMAS-S-EF, and Total Anxiety (EMAS-S-T) (range: 20–100).

EMAS-T: Consists of 60 items measuring anxiety proneness in four situational contexts: (1) social evaluation (EMAS-T-ES), (2) physical danger (EMAS-T-PF), (3) ambiguity (EMAS-T-AM), and (4) daily routine (EMAS-T-DR). Each subscale includes 15 items that are rated on a 5-point Likert scale (1–5) [17], producing non-cumulative scores ranging from 15 to 75 per dimension.

EMAS-P: Evaluates five dimensions of subjective anxiety perception across different situations. In the present study, the EMAS-P dimensions were interpreted within the clinical context of HD to facilitate context-relevant assessment, without structural modification of the instrument: (1) perceived threat (EMAS-P-ES), (2) perceived physical danger (EMAS-P-PF), (3) ambiguity (EMAS-P-AM), (4) perceived danger in routine activities (EMAS-P-DR), and (5) perceived anxiety specific to the testing situation (EMAS-P-A). For the structural check, the following 11 EMAS domain scores were used: EMAS-S-C; EMAS-S-EF; EMAS-T-ES/PF/AM/DR; and EMAS-P-ES/PF/AM/DR/A (T-scores). This approach preserves the original psychometric structure of the EMAS while allowing context-specific interpretation in the HD setting without altering the underlying construct validity of the instrument.

For clarity, the table suffixes denote the EMAS subscales as follows: EMAS-S-C (cognitive), EMAS-S-EF (emotional–physiological), EMAS-S-T (total), EMAS-T-ES (social evaluation), EMAS-T-PF (physical danger), EMAS-T-AM (ambiguity), and EMAS-T-DR (daily routine); EMAS-P-ES/PF/AM/DR correspond to perceived anxiety in the same situational domains, and EMAS-P-A denotes the perceived anxiety specific to the testing situation.

Although the EMAS has been standardized for the Romanian population, evidence for its structural coherence in HD patients is scarce. Therefore, in addition to reliability (internal consistency and temporal stability) and convergent validity, we performed an exploratory structural check in this clinical sample. Given the available dataset structure and sample size, this exploratory factor analysis was conducted at the level of EMAS domain scores (not item-level), using the first administration (T1) to ensure one observation per participant. Item-level factor analysis (EFA/CFA) is generally considered superior for evaluating dimensionality, construct validity, and measurement invariance, as it allows direct assessment of item loadings and cross-loading patterns. However, given the present sample size and domain-aggregated dataset structure, such analyses were not feasible, and the domain-level approach was used as a pragmatic exploratory alternative.

All EMAS scores were interpreted according to Endler’s manual by transforming raw scores into T-scores, adjusted for sex and age intervals [18]. The EMAS, grounded in an interactional model where anxiety represents an outcome of person–situation interactions, is recognized as a highly valid and reliable psychometric instrument [19].

To reduce respondent burden, we did not administer the optional EMAS-SAS module (supplementary social anxiety scale), as social evaluation is already covered by the EMAS-T domain structure and EMAS-SAS was not required for the present aims.

#### 2.4.3. Additional Measures

For external validation, the anxiety subscale of the DASS-21R—adapted and standardized for the Romanian population—was used given its established use in clinical populations and its suitability as a convergent measure of anxiety. Developed by Syd and Peter Lovibond in 1995, it includes seven items that are rated on a 0–5 Likert scale assessing four primary anxiety dimensions: (1) autonomic arousal, (2) musculoskeletal tension, (3) situational anxiety, and (4) subjective anxiety experience, with higher scores indicating greater levels of anxiety [20].

Socioeconomic status (SES) was determined using SES-3, comprising education, occupation, and household income sub-scores, with total scores ranging from 0 to 6: 0–2 = low SES; 3–4 = medium SES; and 5–6 = high SES [21].

### 2.5. Statistical Analysis

Data were analyzed using IBM SPSS Statistics version 26 (IBM Corp., Armonk, NY, USA) in accordance with the EMAS manual guidelines and processed and organized using Microsoft Office 365 (Microsoft Corp., Redmond, WA, USA), and figures were prepared using Adobe InDesign v20.5 (64 bit; Adobe Inc., San Jose, CA, USA). Five major analytic steps were followed:

1. Descriptive statistics:

Mean, standard deviation, and t-values were calculated for comparisons of independent samples.

2. Psychometric characteristics:

Temporal reliability was assessed through test–retest correlations using Pearson’s r coefficient and corresponding participant counts (*n*).

3. Internal consistency:

Internal consistency was evaluated at the domain-score level by computing Cronbach’s alpha for the sets of domain scores within EMAS-T (ES, PF, AM, and DR) and EMAS-P (ES, PF, AM, DR, and A), separately for T1 and T2.

Given that these estimates were derived from aggregated domain scores rather than individual items, Cronbach’s alpha should not be interpreted as a measure of item-level internal consistency. Instead, these values are reported as indicators of inter-domain coherence within a multidimensional framework, reflecting the degree to which related domains show consistent patterns across participants.

4. Test validity:

Convergent validity was assessed through correlations between EMAS and the DASS-21R anxiety subscale using Pearson’s correlation coefficient (r).

5. Intercorrelations between EMAS subscales:

Correlations among EMAS-S, EMAS-T, and EMAS-P were analyzed using Pearson’s r and two-tailed significance (*p*).

Age was handled both as a continuous descriptor and as a categorical variable. While EMAS interpretation uses manual-based normative age intervals, group-wise analyses were performed using collapsed age strata to avoid unstable estimates in very small cells. Age was reported descriptively using 10-year categories consistent with the EMAS manual’s normative intervals. For age-stratified analytic summaries, adjacent decades were collapsed (30–49, 50–59, 60–69, and ≥70 years) to avoid unstable estimates in very small cells (*n* < 10).

Sample size considerations: Prior to analysis, we defined a minimally relevant association for convergent validity as a correlation of at least |r| = 0.30 (small-to-moderate effect). Given the achieved sample size (*n* = 103) and α = 0.05 (two-sided), the study had approximately 90% power to detect correlations of |r| ≥ 0.31 (and 80% power for |r| ≥ 0.27), indicating adequate sensitivity for small-to-moderate associations, while very small effects may remain undetected.

Exploratory factor analysis (EFA) was conducted to provide preliminary evidence of structural coherence for the multidimensional EMAS framework in HD patients. Given the available dataset structure, EFA was performed on domain-level EMAS scores from the first administration (T1), including EMAS-S-C and EMAS-S-EF, the four EMAS-T domains (ES, PF, AM, and DR), and the five EMAS-P domains (ES, PF, AM, DR, and A). Sampling adequacy was evaluated using the Kaiser–Meyer–Olkin (KMO) measure and Bartlett’s test of sphericity. Factors were extracted using principal axis factoring and rotated using direct Oblimin to allow correlated factors. The number of factors was guided by the scree pattern and interpretability, with a three-factor solution examined in line with the theoretical state/trait/perceived architecture. EFA was conducted primarily on T1 domain scores to maintain one record per participant for the main structural exploration. As a sensitivity check, we repeated the same EFA specification on T2 domain scores and compared the interpretive pattern. T2 was otherwise used for the test–retest reliability estimation.

All results were considered statistically significant at *p* < 0.05, with 95% confidence intervals.

### 2.6. Research Ethics

This study was conducted in accordance with the Declaration of Helsinki and approved by the Ethics Committee of the University of Medicine and Pharmacy of Craiova (Approval No. 177/29 October 2021). All participants provided written informed consent prior to inclusion. In cases where severe or unbearable anxiety was identified, participants were ensured a safe environment and offered treatment according to the National Institute for Health and Care Excellence (NICE) guidelines [22].

## 3. Results

A total of 103 patients were included in the final analysis, of whom 48.5% were women (*n* = 50) and 51.5% were men (*n* = 53). The main age group was 60–69 years (*n* = 34; 33.0%). Regarding residence, 43.7% (*n* = 45) lived in urban areas and 56.3% (*n* = 58) in rural areas. Most exclusions occurred prior to completion of the full assessment battery and were primarily related to participant withdrawal due to fatigue and assessment burden. In addition to these exclusions, 29 participants were excluded prior to data entry based on clearly implausible and non-directional EMAS response patterns identified by the administering clinician upon receipt of the questionnaires. As an indirect indicator of response stability in the retained analytical sample, DASS-21R subscale scores demonstrated high temporal consistency over the 4-week interval (Anxiety: r = 0.888, ICC = 0.883; Depression: r = 0.958, ICC = 0.952; Stress: r = 0.928, ICC = 0.927; all *p* < 0.001, *n* = 103). The distribution of T1–T2 differences was approximately symmetric and centered near zero across all three subscales, with 91.3%, 94.2%, and 93.1% of individual differences falling within ±2SD for anxiety, depression, and stress, respectively, supporting the overall temporal coherence of the retained sample.

Educational levels showed that 66.0% had completed primary or middle school, 19.4% attended post-secondary or vocational schools, and 14.6% held university or postgraduate degrees.

Occupationally, the largest subgroup included unskilled laborers, manual workers, and agricultural employees (45.6%), followed by administrative, service, or sales workers (38.8%) and professionals, entrepreneurs, or managers (15.5%) (Table 1).

### 3.1. Gender Differences in Anxiety Scores

Our analysis of the EMAS results revealed that EMAS-S scores were globally higher in women across all subscales.

Specifically, women obtained higher mean scores on the EMAS-S-C (23.98 vs. 17.17), the EMAS-S-EF (21.80 vs. 15.60), and the EMAS-S-T score (45.78 vs. 32.79) compared with men (*p* < 0.001 for all).

EMAS-T scores were also higher in women, although the differences were not statistically significant.

Regarding EMAS-P, men scored higher in most dimensions, except for EMAS-P-AM, where women reported higher anxiety levels (2.78 vs. 2.23).

These findings indicate that anxiety as a state was significantly more intense in women, while trait and perceived anxiety patterns were more balanced across genders (Table 2).

### 3.2. Age-Related Differences

Participant age distribution by 10-year bands is summarized in Table 1. For age-stratified analytic summaries, adjacent decades were collapsed (30–49, 50–59, 60–69, and ≥70 years) to avoid unstable estimates in very small cells (n < 10), and the corresponding descriptive statistics are reported in Table 3.

Across collapsed strata, EMAS-S increased with age, particularly from 50 years onward. Mean EMAS-S-C rose from 14.29 (30–49 years) to 19.40 (50–59 years), 22.44 (60–69 years), and 24.39 (≥70 years), with a similar upward pattern for EMAS-S-EF and total EMAS-S (Table 3). By contrast, EMAS-T and EMAS-P subscales showed comparatively stable values across age strata, without clear age-related gradients (Table 3).

### 3.3. Test–Retest Reliability

Test–retest reliability over the 4-week interval across EMAS subscales (Pearson’s r = 0.837–0.987; ICC = 0.837–0.987; *p* < 0.01) indicated excellent temporal stability in both women and men (Table 4). Pearson correlation coefficients indicated high temporal stability across all subscales (r = 0.90–0.98; *p* < 0.01), confirming excellent test–retest reliability for both genders (Table 4).

### 3.4. Reliability and Temporal Stability

Reliability was evaluated at the total-sample level (*n* = 103). Because EMAS is explicitly multidimensional, internal consistency was summarized at the domain-score level. Inter-domain coherence was good for EMAS-S domains (α = 0.847 at T1; α = 0.835 at T2) and acceptable for EMAS-T domains (α = 0.778 at T1; α = 0.779 at T2). In contrast, coherence across EMAS-P perceived anxiety domains was low (α = 0.192 at T1; α = 0.174 at T2), consistent with the intended context-specific structure of perceived anxiety; therefore, EMAS-P domains were interpreted as distinct situational appraisals rather than as a single homogeneous score.

Temporal stability was examined between the two EMAS administrations performed 4 weeks apart (T1 = I; T2 = II) using test–retest correlations and intraclass correlation coefficients. Across subscales, stability was consistently high (Pearson’s r = 0.837–0.987; ICC = 0.837–0.987), supporting reproducibility of EMAS-derived domain scores over the retest interval. The highest stability was observed for perceived anxiety dimensions (e.g., EMAS-P-ES: r = 0.987; ICC = 0.987), whereas trait anxiety in the physical danger domain showed comparatively lower, yet still acceptable, stability (EMAS-T-PF: r = 0.837; ICC = 0.837).

### 3.5. Exploratory Factor Analysis of EMAS Domain Scores

An exploratory factor analysis (EFA) of the 11 EMAS domain scores at T1 (*n* = 102, listwise) supported factorability (KMO = 0.689; Bartlett’s χ^2^(55) = 348.15, *p* < 0.001). We used principal axis factoring with direct Oblimin rotation (loadings < 0.30 suppressed). Parallel analysis suggested a parsimonious two-factor retention; however, inspection of eigenvalues (λ1 ≈ 3.8, λ2 ≈ 2.6, λ3 ≈ 1.4) and the conceptual interpretability of the solution supported examination of a three-factor structure. The three-factor pattern was interpretable and broadly consistent with the intended framework: the two EMAS-S domains loaded strongly on a State Anxiety component (pattern coefficients ≈ 0.72–1.01), the EMAS-T domains loaded on a Trait/Context component (≈ 0.42–0.99), and the EMAS-P domains loaded on a Perceived Anxiety component (|λ| ≈ 0.41–0.69), with the global perceived anxiety domain approaching the suppression threshold at T1 (λ ≈ 0.30) and the routine-danger domain loading negatively (λ ≈ −0.56), consistent with context-specific appraisal rather than redundancy. Factor loadings were examined at the domain level and showed minimal cross-loading between components, tentatively consistent with a separation between state, trait/context, and perceived anxiety dimensions; however, these findings should be interpreted as exploratory approximations, as item-level analyses would be required to draw firm conclusions about dimensional structure. As a sensitivity check, repeating the same EFA specification on T2 domain scores yielded the same three-component interpretive pattern, with the perceived anxiety domain loading more clearly on the perceived appraisal component. Given the domain-level approach of this analysis, detailed item-level loading matrices are not presented, and the findings should be interpreted as an exploratory structural approximation rather than a formal factorial validation.

### 3.6. Convergent Validity: Correlation with DASS-21R

To assess convergent validity, EMAS scores were correlated with the DASS-21R anxiety subscale using Pearson’s r, and corresponding 95% confidence intervals were estimated (Table 5). The DASS-21R anxiety subscale showed good internal consistency in the present sample (α = 0.863). Overall, correlations between EMAS subscales and DASS-21R anxiety were small and largely non-significant, with two significant associations observed in men: EMAS-T-AM was negatively correlated with DASS-21R anxiety (r = −0.293; *p* = 0.033), while EMAS-P-ES was positively correlated with DASS-21R anxiety (r = 0.273; *p* = 0.048). These findings indicate small, largely non-significant associations between EMAS and DASS-21R anxiety, which limit the strength of convergent validity evidence. The few significant associations were domain-specific and of small magnitude, suggesting only partial overlap between the two instruments.

The precision of the estimated correlations was further examined using 95% confidence intervals calculated via Fisher’s z transformation. In women (*n* = 50), the confidence intervals were generally wide and included zero across all EMAS subscales (e.g., EMAS-S-C: −0.14 to 0.41; EMAS-T-AM: −0.04 to 0.48; EMAS-P-ES: −0.14 to 0.40), supporting the absence of statistically robust associations. In men (*n* = 53), most confidence intervals similarly included zero (e.g., EMAS-S-C: −0.41 to 0.11; EMAS-T-DR: −0.43 to 0.10), with the exception of two subscales: EMAS-T-AM showed a negative association (95% CI: −0.52 to −0.03), and EMAS-P-ES showed a positive association (95% CI: 0.01 to 0.50). Overall, the pattern of confidence intervals supports the interpretation of small and domain-specific effects, consistent with limited but conceptually meaningful overlap between EMAS and DASS-21R anxiety constructs.

### 3.7. Intercorrelations Between EMAS Subscales

The EMAS subscales showed strong positive intercorrelations, confirming that they assess related but distinct aspects of anxiety.

For female participants, the EMAS-S, EMAS-T, and EMAS-P subscales correlated strongly with each other (*r* = 0.46–0.97; *p* < 0.01), indicating coherence among the cognitive, emotional, and situational components of anxiety.

In men, these correlations were similarly positive but slightly lower (*r* = 0.29–0.74; *p* < 0.05), suggesting that, while anxiety dimensions interact, the expression of situational and trait anxiety may differ by gender.

Notably, the EMAS-P subscales exhibited significant cross-links with EMAS-T, particularly between EMAS-P-PF and EMAS-T-AM (*r* = 0.45–0.50; *p* < 0.01), highlighting the connection between perceived physical danger and ambiguity-related trait anxiety.

Overall, these findings support the multidimensional structure and internal coherence of the EMAS model in HD patients (Table 6).

### 3.8. Summary of Findings

In this cohort of 103 HD patients, women showed higher levels of state anxiety than men, particularly in the cognitive and emotional–physiological domains. State anxiety increased with age, with higher values observed from 50 years onward. Over the 4-week retest interval, EMAS domain scores demonstrated high temporal stability (Pearson’s r and ICCs ≈ 0.84–0.99), supporting reproducibility of the measurements. Domain-level internal consistency was good for state anxiety domains and acceptable for trait anxiety domains at both administrations, whereas perceived anxiety domains were interpreted as distinct situational appraisals rather than a single homogeneous score due to low cross-domain coherence. Convergent validity with DASS-21R anxiety was modest and domain-specific, with small correlations overall and only isolated significant associations. Exploratory domain-level factor analyses supported a broadly coherent state/trait/perceived pattern, consistent across both administrations (Table 7).

Overall, the results provide a coherent picture of the psychometric behavior of EMAS in HD patients, including reliability, convergent validity, and preliminary structural consistency across repeated assessments.

## 4. Discussion

### 4.1. Main Findings in Context of the Literature

The present findings provide a structured evaluation of EMAS performance in HD patients, integrating reliability estimates, convergent validity with DASS-21R, and exploratory structural patterns across two assessment points. From a clinical perspective, this multidimensional approach may assist clinicians in distinguishing between transient situational anxiety and more stable vulnerability patterns, thereby supporting more targeted psychological and behavioral interventions. It may also facilitate better communication within multidisciplinary teams by providing a structured profile of anxiety dimensions relevant to treatment adherence, coping strategies, and overall patient management.

Previous research assessing anxiety in CKD and HD populations has often relied on simpler or more general screening tools. Commonly used instruments include the BAI, HADS, and STAI. These tools, while effective in clinical practice, primarily capture limited dimensions of anxiety—often as a unitary construct—without differentiating situational, personality-related, and perceptual components.

In contrast, the EMAS offers a multidimensional framework for understanding anxiety. It distinguishes between state anxiety (temporary emotional reactivity), trait anxiety (individual disposition), and perceived anxiety (subjective evaluation of threat in specific contexts). This multidimensional approach allows for a more nuanced assessment of psychological responses in HD patients—individuals who face continuous medical stressors, lifestyle restrictions, and existential uncertainty associated with renal replacement therapy [23,24,25,26].

Our exploratory domain-level EFA yielded a pattern broadly consistent with the conceptual triad in HD patients: the two EMAS-S domains clustered on a State Anxiety component, the EMAS-T domains on a Trait/Context component, and the EMAS-P domains on a Perceived Anxiety component, albeit with some context-specific (including negative) loadings consistent with situational appraisal rather than redundancy. These findings are preliminary and exploratory in nature; the domain-level approach is not a substitute for item-level EFA/CFA, and the observed pattern should not be interpreted as structural confirmation of dimensionality. Item-level analyses on larger samples would be required to draw firm conclusions about the factorial structure of EMAS in HD populations.

Despite the clinical utility of the EMAS, our review of the international literature found no prior clinical studies applying it to HD populations. Given that, based on our current knowledge, EMAS has not been previously used in HD populations, the present results provide initial, context-specific benchmarks that should be confirmed through replication and cross-cultural comparisons. A likely reason lies in the instrument’s complexity and length—it comprises 85 items, far exceeding the number used by tools such as the BAI (21 items), HADS (14 items), or STAI (20 items). For HD patients, whose levels of fatigue and concentration may fluctuate during dialysis, lengthy questionnaires can be challenging to complete, explaining the relatively high dropout rate in our sample.

Nevertheless, the EMAS remains a highly valuable research instrument, providing detailed quantitative insights into both cognitive and physiological dimensions of anxiety. It is important to emphasize that the EMAS is not a diagnostic instrument but a psychometric research tool. It measures the level and type of anxiety, yielding norm-referenced scores that facilitate complex and detailed analysis rather than categorical diagnosis. This makes EMAS particularly suitable for research exploring the interaction between personality traits, cognition, and situational stress.

From a clinical feasibility perspective, the full 85-item EMAS battery is not suitable for routine screening in dialysis settings, given the significant respondent burden it imposes on a population already affected by fatigue and reduced concentration capacity. In our cohort, voluntary withdrawal due to assessment burden was the most frequent reason for non-completion (*n* = 60), underscoring the importance of instrument length as a practical barrier. The present study was designed as a research-oriented psychometric evaluation, and EMAS should be regarded as a multidimensional profiling tool for use in structured research contexts rather than in routine clinical practice. Future work should focus on developing and validating abbreviated EMAS versions that preserve the state/trait/perceived architecture while substantially reducing respondent burden, thereby supporting feasibility in HD clinical settings.

When compared to the STAI, both scales share the fundamental distinction between state and trait anxiety [27]. We referenced the STAI because it is the most widely used state–trait framework and provides a familiar conceptual anchor for interpreting EMAS outputs. However, EMAS is grounded in an interactional person–situation model and extends beyond global state/trait scores by differentiating trait anxiety across situational domains (e.g., social evaluation, physical danger, ambiguity, and daily routine) and by incorporating perceived anxiety. This added granularity is potentially relevant in HD, where anxiety may be driven by specific treatment-related contexts rather than a single unitary construct. In clinical HD samples, this state–trait separation has proven practically informative; for example, in a cohort of 105 HD patients assessed with the STAI, women showed significantly higher state anxiety than men, and state/trait levels varied with sociodemographic and behavioral factors, underscoring the value of distinguishing transient reactivity from more stable vulnerability.

Compared with the DASS-21, which assesses depression, anxiety, and stress simultaneously, the EMAS focuses exclusively on anxiety within a multidimensional, person-situation framework. Previous research has reported moderate-to-strong convergent validity between the EMAS and the anxiety subscale of the DASS-21 (r = 0.70–0.85), supporting their shared anxiety-related construct. In the present study, the DASS-21R anxiety subscale demonstrated good internal consistency (α = 0.863); however, correlations between EMAS subscales and the DASS-21R anxiety subscale were generally modest. These modest associations should be interpreted cautiously, as they provide only limited support for convergent validity. This pattern is consistent with the instruments’ differing emphases: while the DASS-21R captures general anxiety symptomatology over a brief timeframe [28,29], EMAS operationalizes a multidimensional framework incorporating trait domains and perceived anxiety [19]. The limited overlap observed in our results also reflects the restricted strength of the convergent validity evidence obtained in this sample, while at the same time suggesting that EMAS captures anxiety facets not fully represented by brief screening scales.

The HADS is frequently employed in hospital settings for detecting anxiety and depression in medical inpatients [30]. While this tool provides clear clinical thresholds for identifying anxious symptomatology, the EMAS offers a more detailed and multidimensional profile of expressions of anxiety, which is valuable for understanding the interplay between somatic and psychological factors in chronically ill patients.

Consistent with the pragmatic preference for brief screening in dialysis care, an HD study validating short anxiety screening tools reported good performance for both the BAI and the HADS-Anxiety subscale, while noting that shorter instruments may reduce respondent burden and facilitate routine implementation in clinical settings.

In comparison, the BAI predominantly measures somatic and cognitive manifestations of anxiety (e.g., palpitations, dizziness, and tension) [31], whereas the EMAS also explores psychological processes and situational triggers. Correlations between the EMAS and BAI are generally moderate (r = 0.55–0.70), indicating that the two scales measure complementary aspects of anxiety—the BAI emphasizes symptoms, while the EMAS focuses on context and perception.

Previous studies have found similar patterns when comparing the EMAS with the STAI, suggesting that both tools assess overlapping constructs. A 2025 study involving 1177 non-clinical participants reported high convergence, with excellent internal consistency and significant correlations between the STAI and both the EMAS-S (r = 0.773; *p* < 0.001) and EMAS-T (r = 0.691; *p* < 0.001) [32]. Older research also confirmed the EMAS’s ability to clearly differentiate between state anxiety, which is transient and situation-dependent, and trait anxiety, a more stable personality characteristic [19].

Most research teams recommend periodic screening for anxiety among HD patients—at initiation of therapy and regular intervals thereafter—since anxiety symptoms may fluctuate over time due to medical exacerbations, functional decline, or changes in social/family support [33].

The literature supports a range of psychosocial and pharmacological interventions to mitigate anxiety in dialysis patients. These include the following:

(1) Cognitive behavioral therapy (CBT) and other structured psychotherapeutic approaches [12,34]; (2) symptom-targeted interventions, delivered during dialysis sessions [35,36]; (3) relaxation techniques, such as the Benson relaxation protocol; (4) spiritual counseling and family support interventions [37,38]; and (5) pharmacological management, involving antidepressants or anxiolytics, with dosage adjustments and careful monitoring for renal safety and drug interactions [39].

Meta-analyses and randomized controlled trials have demonstrated that these psychosocial and relaxation-based interventions can significantly reduce anxiety symptoms and improve patients’ quality of life [40,41]. However, study quality and methodological consistency vary widely, highlighting the need for large-scale, longitudinal, and multicenter studies with robust designs and standardized instruments.

The results of this study emphasize the necessity of integrating multidimensional psychological assessments into the clinical management of HD patients. Anxiety—when unrecognized or untreated—can affect adherence, clinical outcomes, and overall quality of life.

Collaboration between nephrologists, psychiatrists, and psychologists is therefore essential for establishing effective screening, early detection, and treatment pathways. As identified in the literature, future research should focus on (1) the standardization of psychometric instruments used in dialysis research, (2) the implementation of longitudinal and randomized clinical trials with multiple endpoints (mortality, hospitalization rates, therapeutic adherence), and (3) the incorporation of brief, targeted interventions that can be delivered during HD sessions to address acute anxiety and stress [42,43,44,45,46,47,48,49].

Building multidisciplinary teams that combine medical and psychological expertise remains key to improving both mental health and treatment adherence among patients with CKD who are undergoing dialysis.

This integrative perspective reflects the principles of psychonephrology, a discipline bridging nephrology and psychiatry to optimize holistic patient care [50,51]. Psychonephrology refers to an integrated biopsychosocial approach embedded in kidney care, emphasizing systematic screening for psychological distress, timely referral to mental health services, and coordinated interdisciplinary management. In HD settings, this framework supports feasible screening pathways (brief tools for routine use, with escalation when needed) and targeted interventions aligned with symptom severity, functional impairment, and treatment adherence. From a research perspective, it also encourages the use of validated instruments and standardized reporting to facilitate comparability across dialysis cohorts [50,51].

### 4.2. Limitations

Several limitations should be acknowledged. First, the single-center design and the sample size (*n* = 103) limit external generalizability and robust subgroup inference. To address statistical sensitivity for the convergent validity objective, we conducted a correlation-based sensitivity power analysis: with *n* = 103 and α = 0.05 (two-sided), the minimum detectable correlation is approximately |r| ≥ 0.31 for 90% power (and |r| ≥ 0.27 for 80% power), indicating adequate sensitivity for small-to-moderate associations, while very small effects may remain undetected.

Second, our structural assessment was exploratory and conducted at the level of EMAS domain scores rather than item-level responses. While this provides a pragmatic check of the intended state/trait/perceived architecture in a clinical HD sample, it does not replace item-level EFA/CFA and should be interpreted as preliminary. Larger multicenter studies with item-level data are warranted to test dimensional stability more rigorously (including measurement invariance) and to inform feasibility-oriented shortening. In addition, internal consistency estimates (Cronbach’s alpha) were computed at the level of aggregated domain scores rather than individual items. Consequently, these values should not be interpreted as indicators of item-level internal consistency but rather as exploratory measures of inter-domain coherence within a multidimensional construct.

Third, EMAS is a relatively long instrument (85 items), and the overall assessment battery (EMAS plus MMSE and DASS-21R) may increase respondent burden in HD patients, potentially inducing fatigue and contributing to attrition. In our cohort, voluntary withdrawal due to fatigue/lengthy testing time was substantial (*n* = 60), which may introduce selection bias if completers differ systematically from those who withdrew. Because a substantial proportion of exclusions occurred before completion of the full assessment battery, demographic and clinical comparisons between included and excluded participants were not feasible. This limits the ability to formally assess potential selection bias and should be considered when interpreting the generalizability of the findings.

Accordingly, we interpret feasibility and generalizability cautiously and do not propose EMAS for routine dialysis screening; rather, it is positioned as a multidimensional profiling instrument when a detailed assessment is justified.

Fourth, the study included two EMAS administrations (4-week interval) primarily to estimate test–retest reliability and was not designed to model short-term anxiety dynamics, which would require more frequent repeated measurements.

An additional methodological consideration concerns the exclusion of 29 participants due to implausible response patterns across EMAS administrations. These cases were identified clinically at the point of questionnaire collection, prior to data entry, based on the administering clinician’s judgment that the observed patterns were inconsistent with any plausible psychological trajectory over the 4-week interval. Because the original questionnaires were not retained, retrospective quantification of T1–T2 discrepancies for these participants was not feasible, and a formal sensitivity analysis including them could not be performed. We acknowledge that the absence of a pre-specified numerical exclusion threshold and the inability to compare excluded with retained participants represent limitations that reduce methodological transparency. As an indirect indicator of response stability in the analytical sample, DASS-21R subscale scores demonstrated high temporal consistency over the 4-week interval (r = 0.888–0.958; ICC = 0.883–0.952; all *p* < 0.001), suggesting that the retained sample exhibits coherent repeated-response patterns. Future studies should pre-specify quantitative criteria for response quality screening prior to data collection.

Another limitation relates to the contextual framing of the EMAS-P component for the HD setting. Although no structural modifications were performed, the interpretation of perceived anxiety may be influenced by the clinical context. As a formal validation of this contextual application was not conducted, these findings should be interpreted with caution and confirmed in future studies.

Finally, comparative evidence for EMAS in HD cohorts remains limited because the instrument has seldom been applied in dialysis populations; therefore, our findings—including the exploratory domain-level factor pattern—should be interpreted as preliminary and warrant replication and benchmarking in larger multi-center HD samples with item-level structural testing.

## 5. Conclusions

Anxiety represents a complex, multidimensional construct with a high prevalence among patients undergoing HD.

In this study, anxiety levels were influenced by both gender and age, with female and older patients exhibiting higher state anxiety scores.

EMAS demonstrated good reliability at the domain level and provided limited but domain-specific evidence of convergent validity for the multidimensional assessment of anxiety in this HD cohort. The instrument’s ability to distinguish between state, trait, and perceived anxiety enables a deeper understanding of the psychological mechanisms associated with chronic illness and renal replacement therapy.

Given these findings, the integration of psychological assessment is warranted in dialysis centers; however, EMAS should be considered primarily as a research-oriented multidimensional profiling instrument rather than a tool for routine screening, due to its length and complexity.

Integrating psychometric evaluation within multidisciplinary care—linking nephrology, psychiatry, and psychology—can substantially improve patient outcomes, quality of life, and treatment adherence.

Future research should further investigate multiple facets of anxiety, inform the development of shorter EMAS versions, and support tailored interventions targeting specific anxiety dimensions identified through multidimensional assessments.

These findings should be interpreted cautiously, as the structural analyses were exploratory and conducted at the domain level and require confirmation in larger samples with item-level validation.

## Figures and Tables

**Table 1:** Demographic characteristics of the participants.

Parameter	Value	Frequency	Percent
Age	30–39 years	6	5.8
	40–49 years	15	14.6
	50–59 years	25	24.3
	60–69 years	34	33.0
	70–79 years	20	19.4
	≥80 years	3	2.9
Residence	Urban	45	43.7
	Rural	58	56.3
Studies	Primary school, middle school	68	66.0
	Highschool, post-secondary/vocational school	20	19.4
	University/post-university studies	15	14.6
Occupation	Unskilled labor, manual labor, agricultural labor	47	45.6
	Administrative officer, services, sales technician	40	38.8
	Professional, entrepreneur, manager	16	15.5
Household income	Medium threshold (50–100% of the median line)	30	29.1
	High threshold (>median line or over 2× minimum wage in the economy)	73	70.9
Socioeconomic status level	Low	29	28.1
	Medium	62	60.2
	High	12	11.7
	Total	103	100.0

**Table 2:** Gender differences—EMAS.

		Women			Men				
		N	Mean	Std. Deviation	N	Mean	Std. Deviation	Std. Error Mean	t
EMAS-S	EMAS-S-C	50	23.98	10.596	53	17.17	5.560	0.764	4.11752 **
	EMAS-S-EF	50	21.80	9.806	53	15.60	5.576	0.766	3.97041 **
	EMAS-S-T	50	45.78	19.659	53	32.79	8.198	1.126	4.42043 **
EMAS-T	EMAS-T-ES	50	38.56	13.241	53	37.09	11.590	1.592	0.59867
	EMAS-T-PF	50	54.14	12.102	53	49.45	13.321	1.830	1.86557
	EMAS-T-AM	50	35.10	12.604	53	34.34	15.169	2.084	0.27581
	EMAS-T-DR	50	28.64	13.899	53	29.81	11.474	1.576	0.46751
EMAS-P	EMAS-P-ES	50	2.66	1.611	53	2.96	1.467	0.202	0.99637
	EMAS-P-PF	50	1.78	1.166	53	2.23	1.310	0.180	1.82277
	EMAS-P-AM	50	2.78	1.620	53	2.23	1.310	0.180	1.91187
	EMAS-P-DR	50	3.72	1.294	53	3.77	1.235	0.170	0.21503
	EMAS-P-A	50	1.68	1.203	53	1.74	1.003	0.138	0.25651

** *p* < 0.001.

**Table 3:** EMAS subscale descriptive statistics by collapsed age strata (mean, SD, and SEM).

		Age	N	Mean	Std. Deviation	Std. Error Mean
EMAS-S	EMAS-S-C	30–49 years	21	14.29	4.337	0.946
		50–59 years	25	19.40	8.981	1.796
		60–69 years	34	22.44	7.632	1.309
		≥70 years	23	24.39	11.179	2.331
	EMAS-S-EF	30–49 years	21	15.57	5.741	1.253
		50–59 years	25	18.48	9.125	1.825
		60–69 years	34	18.71	7.803	1.338
		≥70 years	23	21.39	10.175	2.122
	EMAS-S-T	30–49 years	21	29.91	8.983	1.960
		50–59 years	25	37.88	16.574	3.315
		60–69 years	34	41.15	13.998	2.401
		≥70 years	23	45.78	20.355	4.244
EMAS-T	EMAS-T-ES	30–49 years	21	41.67	12.726	2.777
		50–59 years	25	33.28	8.218	1.644
		60–69 years	34	37.65	13.602	2.333
		≥70 years	23	39.43	13.104	2.732
	EMAS-T-PF	30–49 years	21	55.05	10.966	2.393
		50–59 years	25	50.80	15.362	3.072
		60–69 years	34	49.29	12.937	2.219
		≥70 years	23	53.30	11.342	2.365
	EMAS-T-AM	30–49 years	21	38.38	14.269	3.114
		50–59 years	25	30.72	10.699	2.140
		60–69 years	34	34.38	15.653	2.685
		≥70 years	23	36.17	13.703	2.857
	EMAS-T-DR	30–49 years	21	27.57	11.016	2.404
		50–59 years	25	27.48	10.149	2.030
		60–69 years	34	29.24	13.629	2.337
		≥70 years	23	32.70	14.926	3.112
EMAS-P	EMAS-P-ES	30–49 years	21	2.90	1.409	0.307
		50–59 years	25	2.76	1.739	0.348
		60–69 years	34	2.56	1.418	0.243
		≥70 years	23	3.17	1.639	0.342
	EMAS-P-PF	30–49 years	21	2.00	1.142	0.249
		50–59 years	25	2.12	1.333	0.267
		60–69 years	34	1.62	0.985	0.169
		≥70 years	23	2.48	1.468	0.306
	EMAS-P-AM	30–49 years	21	2.33	1.392	0.304
		50–59 years	25	2.64	1.578	0.316
		60–69 years	34	2.41	1.598	0.274
		≥70 years	23	2.61	1.377	0.287
	EMAS-P-DR	30–49 years	21	3.71	1.228	0.268
		50–59 years	25	3.60	1.354	0.271
		60–69 years	34	4.09	1.240	0.213
		≥70 years	23	3.43	1.137	0.237
	EMAS-P-A	30–49 years	21	1.67	1.030	0.225
		50–59 years	25	1.80	1.190	0.238
		60–69 years	34	1.62	1.045	0.179
		≥70 years	23	1.78	1.185	0.247

**Table 4:** EMAS test–retest reliability.

			Women				Men			
			N	Mean	Std. Deviation	r	N	Mean	Std. Deviation	r
EMAS-S	EMAS-S-C	test	50	23.66	9.944	0.904 **	53	16.43	5.344	0.904 **
		retest	50	23.98	10.596	0.904 **	53	17.17	5.560	0.904 **
	EMAS-S-EF	test	50	20.40	8.894	0.964 **	53	15.11	4.814	0.908 **
		retest	50	21.80	9.806	0.964 **	53	15.60	5.576	0.908 **
	EMAS-S-T	test	50	44.00	17.884	0.981 **	53	31.55	8.168	0.949 **
		retest	50	45.78	19.659	0.981 **	53	32.79	8.198	0.949 **
EMAS-T	EMAS-T-ES	test	50	37.12	12.962	0.975 **	53	35.42	11.374	0.975 **
		retest	50	38.56	13.241	0.975 **	53	37.09	11.590	0.975 **
	EMAS-T-PF	test	50	54.58	11.680	0.961 **	53	48.77	13.272	0.733 **
		retest	50	54.14	12.102	0.961 **	53	49.45	13.321	0.733 **
	EMAS-T-AM	test	50	32.54	14.607	0.950 **	53	32.57	14.720	0.980 **
		retest	50	35.10	12.604	0.950 **	53	34.34	15.169	0.980 **
	EMAS-T-DR	test	50	27.24	13.313	0.969 **	53	27.45	11.436	0.928 **
		retest	50	28.64	13.899	0.969 **	53	29.81	11.474	0.928 **
EMAS-P	EMAS-P-ES	test	50	2.52	1.619	0.976 **	53	2.96	1.467	1.000 **
		retest	50	2.66	1.611	0.976 **	53	2.96	1.467	1.000 **
	EMAS-P-PF	test	50	1.72	1.126	0.901 **	53	2.28	1.321	0.873 **
		retest	50	1.78	1.166	0.901 **	53	2.23	1.310	0.873 **
	EMAS-P-AM	test	50	2.82	1.612	0.938 **	53	2.21	1.306	0.972 **
		retest	50	2.78	1.620	0.938 **	53	2.23	1.310	0.972 **
	EMAS-P-DR	test	50	3.64	1.367	0.911 **	53	3.87	1.127	0.918 **
		retest	50	3.72	1.294	0.911 **	53	3.77	1.235	0.918 **
	EMAS-P-A	test	50	1.60	1.107	0.883 **	53	1.74	1.003	1.000 **
		retest	50	1.68	1.203	0.883 **	53	1.74	1.003	1.000 **

** Correlation is significant at the 0.01 level (2-tailed).

**Table 5:** Convergent validity between DASS-21 R, anxiety subscale, and EMAS.

		Women			Men		
			Anxiety			Anxiety	
		N	r	*p*	N	r	*p*
EMAS-S	EMAS-S-C	50	0.144	0.318	53	−0.159	0.257
	EMAS-S-EF	50	0.140	0.333	53	−0.031	0.825
	EMAS-S-T	50	0.147	0.307	53	−0.130	0.355
EMAS-T	EMAS-T-ES	50	0.175	0.225	53	−0.134	0.339
	EMAS-T-PF	50	0.016	0.911	53	−0.037	0.794
	EMAS-T-AM	50	0.240	0.093	53	−0.293 *	0.033
	EMAS-T-DR	50	0.066	0.651	53	−0.174	0.212
EMAS-P	EMAS-P-ES	50	0.134	0.352	53	0.273 *	0.048
	EMAS-P-PF	50	0.079	0.585	53	−0.031	0.826
	EMAS-P-AM	50	0.010	0.943	53	0.064	0.650
	EMAS-P-DR	50	−0.114	0.431	53	−0.016	0.907
	EMAS-P-A	50	0.037	0.796	53	−0.049	0.727

* Correlation is significant at the 0.05 level (2-tailed).

**Table 6:** Intercorrelations between EMAS subscales.

			EMAS									
			EMAS-S									
			EMAS-T-C		EMAS-T-ES		EMAS-T-Total					
		G/N	r	*p*	r	*p*	r	*p*				
EMAS-S	EMAS-S-C	F/50	1		0.857 **	0.000	0.966 **	0.000				
		M/53	1		0.083	0.552	0.735 **	0.000				
	EMAS-S-EF	F/50	0.857 **	0.000	1		0.961 **	0.000				
		M/53	0.083	0.552	1		0.737 **	0.000				
	EMAS-S-T	F/50	0.966 **	0.000	0.961 **	0.000	1					
		M/53	0.735 **	0.000	0.737 **	0.000	1					
EMAS-T	EMAS-T-ES	F/50	0.101	0.486	0.131	0.365	0.120	0.408				
		M/53	0.045	0.748	0.140	0.316	0.125	0.374				
	EMAS-T-PF	F/50	0.148	0.305	0.196	0.171	0.178	0.217				
		M/53	−0.128	0.362	0.018	0.900	−0.073	0.605				
	EMAS-T-AM	F/50	0.099	0.495	0.043	0.766	0.075	0.606				
		M/53	−0.058	0.682	0.092	0.514	0.023	0.873				
	EMAS-T-DR	F/50	0.461 **	0.001	0.344 *	0.015	0.420 **	0.002				
		M/53	0.032	0.821	0.119	0.396	0.100	0.478				
EMAS-P	EMAS-P-ES	F/50	0.049	0.737	0.018	0.904	0.035	0.810				
		M/53	−0.068	0.631	0.170	0.224	0.066	0.636				
	EMAS-P-PF	F/50	0.343 *	0.015	0.301 *	0.033	0.335 *	0.017				
		M/53	0.090	0.523	0.115	0.412	0.137	0.328				
	EMAS-P-AM	F/50	0.190	0.186	0.284 *	0.046	0.244	0.088				
		M/53	−0.108	0.440	0.152	0.277	0.028	0.844				
	EMAS-P-DR	F/50	−0.121	0.403	−0.019	0.896	−0.075	0.606				
		M/53	−0.070	0.619	−0.200	0.150	−0.181	0.194				
	EMAS-P-A	F/50	0.022	0.880	0.038	0.795	0.031	0.833				
		M/53	−0.019	0.890	0.356 **	0.009	0.227	0.102				
			**EMAS-T**									
		**G/N**	**EMAS-T-ES**		**EMAS-T-PF**		**EMAS-T-AM**		**EMAS-T-DR**			
EMAS-S	EMAS-S-C	F/50	r	*p*	r	*p*	r	*p*	r	*p*		
		M/53	0.101	0.486	0.148	0.305	0.099	0.495	0.461 **	0.001		
	EMAS-S-EF	F/50	0.045	0.748	−0.128	0.362	−0.058	0.682	0.032	0.821		
		M/53	0.131	0.365	0.196	0.171	0.043	0.766	0.344 *	0.015		
	EMAS-S-T	F/50	0.140	0.316	0.018	0.900	0.092	0.514	0.119	0.396		
		M/53	0.120	0.408	0.178	0.217	0.075	0.606	0.420 **	0.002		
EMAS-T	EMAS-T-ES	F/50	0.125	0.374	−0.073	0.605	0.023	0.873	0.100	0.478		
		M/53	1		0.296 *	0.037	0.598 **	0.000	0.288 *	0.042		
	EMAS-T-PF	F/50	1		0.543 **	0.000	0.732 **	0.000	0.568 **	0.000		
		M/53	0.296 *	0.037	1		0.344 *	0.014	0.282 *	0.048		
	EMAS-T-AM	F/50	0.543 **	0.000	1		0.556 **	0.000	0.484 **	0.000		
		M/53	0.598 **	0.000	0.344 *	0.014	1		0.372 **	0.008		
	EMAS-T-DR	F/50	0.732 **	0.000	0.556 **	0.000	1		0.639 **	0.000		
		M/53	0.288 *	0.042	0.282 *	0.048	0.372 **	0.008	1			
EMAS-P	EMAS-P-ES	F/50	0.568 **	0.000	0.484 **	0.000	0.639 **	0.000	1			
		M/53	0.099	0.494	0.323 *	0.022	0.241	0.092	−0.017	0.909		
	EMAS-P-PF	F/50	0.336 *	0.014	0.158	0.257	0.273 *	0.048	0.346 *	0.011		
		M/53	0.128	0.374	0.297 *	0.036	0.215	0.133	0.305 *	0.031		
	EMAS-P-AM	F/50	0.323 *	0.018	0.186	0.183	0.218	0.118	0.327 *	0.017		
		M/53	0.435 **	0.002	0.276	0.052	0.234	0.102	0.286 *	0.044		
	EMAS-P-DR	F/50	0.251	0.070	0.202	0.146	0.249	0.073	0.429 **	0.001		
		M/53	−0.276	0.052	−0.377 **	0.007	−0.404 **	0.004	−0.238	0.096		
	EMAS-P-A	F/50	−0.103	0.462	−0.123	0.379	−0.103	0.465	−0.224	0.106		
		M/53	0.234	0.101	−0.061	0.672	−0.057	0.694	0.136	0.347		
			0.141	0.313	−0.053	0.707	0.150	0.283	0.315 *	0.022		
			**EMAS-P**									
			**EMAS-P-ES**		**EMAS-P-PF**		**EMAS-P-AM**		**EMAS-P-DR**		**EMAS-P-A**	
		**G/N**	**r**	***p***	**r**	***p***	**r**	***p***	**r**	***p***	**r**	***p***
EMAS-S	EMAS-S-C	F/50	0.049	0.737	0.343 *	0.015	0.190	0.186	−0.121	0.403	0.022	0.880
		M/53	−0.068	0.631	0.090	0.523	−0.108	0.440	−0.070	0.619	−0.019	0.890
	EMAS-S-EF	F/50	0.018	0.904	0.301 *	0.033	0.284 *	0.046	−0.019	0.896	0.038	0.795
		M/53	0.170	0.224	0.115	0.412	0.152	0.277	−0.200	0.150	0.356 **	0.009
	EMAS-S- T	F/50	0.035	0.810	0.335 *	0.017	0.244	0.088	−0.075	0.606	0.031	0.833
		M/53	0.066	0.636	0.137	0.328	0.028	0.844	−0.181	0.194	0.227	0.102
EMAS-T	EMAS-T-ES	F/50	0.099	0.494	0.128	0.374	0.435 **	0.002	−0.276	0.052	0.234	0.101
		M/53	0.336 *	0.014	0.323 *	0.018	0.251	0.070	−0.103	0.462	0.141	0.313
	EMAS-T-PF	F/50	0.323 *	0.022	0.297 *	0.036	0.276	0.052	−0.377 **	0.007	−0.061	0.672
		M/53	0.158	0.257	0.186	0.183	0.202	0.146	−0.123	0.379	−0.053	0.707
	EMAS-T-AM	F/50	0.241	0.092	0.215	0.133	0.234	0.102	−0.404 **	0.004	−0.057	0.694
		M/53	0.273 *	0.048	0.218	0.118	0.249	0.073	−0.103	0.465	0.150	0.283
	EMAS-T-DR	F/50	−0.017	0.909	0.305 *	0.031	0.286 *	0.044	−0.238	0.096	0.136	0.347
		M/53	0.346 *	0.011	0.327 *	0.017	0.429 **	0.001	−0.224	0.106	0.315 *	0.022
EMAS-P	EMAS-P-ES	F/50	1		0.090	0.535	0.033	0.818	−0.340 *	0.016	0.048	0.741
		M/53	1		0.455 **	0.001	0.255	0.066	−0.238	0.086	0.451 **	0.001
	EMAS-P-PF	F/50	0.090	0.535	1		0.428 **	0.002	−0.475 **	0.000	0.182	0.207
		M/53	0.455 **	0.001	1		0.507 **	0.000	−0.372 **	0.006	0.529 **	0.000
	EMAS-P-AM	F/50	0.033	0.818	0.428 **	0.002	1		−0.497 **	0.000	0.319 *	0.024
		M/53	0.255	0.066	0.507 **	0.000	1		−0.443 **	0.001	0.456 **	0.001
	EMAS-P-DR	F/50	−0.340 *	0.016	−0.475 **	0.000	−0.497 **	0.000	1		−0.177	0.220
		M/53	−0.238	0.086	−0.372 **	0.006	−0.443 **	0.001	1		−0.205	0.142
	EMAS-P-A	F/50	0.048	0.741	0.182	0.207	0.319 *	0.024	−0.177	0.220	1	
		M/53	0.451 **	0.001	0.529 **	0.000	0.456 **	0.001	−0.205	0.142	1	

**. Correlation is significant at the 0.01 level (2-tailed) *. Correlation is significant at the 0.05 level (2-tailed).

**Table 7:** Summary of psychometric indices for EMAS domain scores and DASS-21R anxiety subscale (*n* = 103).

Scale	Component	Cronbach’s α (T1)	Cronbach’s α (T2)	Test–retest r (Range)	ICC (Range)	Convergent Validity r with DASS-21R
EMAS-S	State anxiety domains	0.847	0.835	0.904–0.981	0.904–0.981	−0.159 to 0.147 (ns)
EMAS-T	Trait anxiety domains	0.778	0.779	0.733–0.980	0.733–0.980	−0.293 * to 0.175 (ns)
EMAS-P	Perceived anxiety domains	0.192	0.174	0.873–1.000	0.873–1.000	−0.114 to 0.273 *
DASS-21R	Anxiety subscale	0.863	-	0.888	0.883	—

ns = non-significant; * *p* < 0.05. Cronbach’s α for EMAS computed at domain-score level (inter-domain coherence); for DASS-21R computed at item level. Test–retest r and ICC reported across women and men combined.

## Data Availability

The data presented in this study are available from the corresponding author upon request. The data are not publicly available due to privacy restrictions.

## Abbreviations

The following abbreviations are used in this manuscript:

BAI	Beck Anxiety Inventory
CBT	Cognitive Behavioral Therapy
CKD	Chronic Kidney Disease
DASS-21R	Depression, Anxiety, and Stress Scale-Revised (21 Items)
DSM-5-TR	Diagnostic and Statistical Manual of Mental Disorders, Fifth Edition, Text Revision
EMAS	Endler Multidimensional Anxiety Scales
EMAS-S	State Anxiety Scale of EMAS
EMAS-S-C	Cognitive State Anxiety Subscale
EMAS-S-EF	Emotional–Physiological State Anxiety Subscale
EMAS-S-T	Total Score EMAS-S Scale
EMAS-T	Trait Anxiety Scale of EMAS
EMAS-T-AM	Trait Anxiety—Ambiguity Subscale
EMAS-T-DR	Trait Anxiety—Daily Routine Subscale
EMAS-T-ES	Trait Anxiety—Social Evaluation Subscale
EMAS-T-PF	Trait Anxiety—Physical Danger Subscale
EMAS-P	Perceived Anxiety Scale of EMAS
EMAS-P-A	Perceived Anxiety—Testing Situation Subscale
EMAS-P-AM	Perceived Anxiety—Ambiguity Subscale
EMAS-P-DR	Perceived Anxiety—Daily Routine Subscale
EMAS-P-ES	Perceived Anxiety—Social Evaluation Subscale
EMAS-P-PF	Perceived Anxiety—Physical Danger Subscale
HADS	Hospital Anxiety and Depression Scale
HD	Hemodialysis
KDIGO	Kidney Disease: Improving Global Outcomes
MMSE	Mini-Mental State Examination
NICE	National Institute for Health and Care Excellence
SES	Socioeconomic Status
SES-3	Three-Dimensional Socioeconomic Status Scale
SPSS	Statistical Package for the Social Sciences
STAI	State–Trait Anxiety Inventory
WHO	World Health Organization
